# Opportunities and dilemmas of *in vitro* nano neural electrodes

**DOI:** 10.1039/c9ra08917a

**Published:** 2019-12-24

**Authors:** Yu Wu, Haowen Chen, Liang Guo

**Affiliations:** Department of Electrical and Computer Engineering, The Ohio State University Columbus OH USA guo.725@osu.edu

## Abstract

Developing electrophysiological platforms to capture electrical activities of neurons and exert modulatory stimuli lays the foundation for many neuroscience-related disciplines, including the neuron–machine interface, neuroprosthesis, and mapping of brain circuitry. Intrinsically more advantageous than genetic and chemical neuronal probes, electrical interfaces directly target the fundamental driving force—transmembrane currents—behind the complicated and diverse neuronal signals, allowing for the discovery of neural computational mechanisms of the most accurate extent. Furthermore, establishing electrical access to neurons is so far the most promising solution to integrate large-scale, high-speed modern electronics with neurons that are highly dynamic and adaptive. Over the evolution of electrode-based electrophysiologies, there has long been a trade-off in terms of precision, invasiveness, and parallel access due to limitations in fabrication techniques and insufficient understanding of membrane–electrode interactions. On the one hand, intracellular platforms based on patch clamps and sharp electrodes suffer from acute cellular damage, fluid diffusion, and labor-intensive micromanipulation, with little room for parallel recordings. On the other hand, conventional extracellular microelectrode arrays cannot detect from subcellular compartments or capture subthreshold membrane potentials because of the large electrode size and poor seal resistance, making it impossible to depict a comprehensive picture of a neuron's electrical activities. Recently, the application of nanotechnology on neuronal electrophysiology has brought about a promising solution to mitigate these conflicts on a single chip. In particular, three dimensional nanostructures of 10–100 nm in diameter are naturally fit to achieve the purpose of precise and localized interrogations. Engineering them into vertical nanoprobes bound on planar substrates resulted in excellent membrane–electrode seals and high-density electrode distribution. There is no doubt that 3D vertical nanoelectrodes have achieved a fundamental milestone in terms of high precision, low invasiveness, and parallel recording at the neuron–electrode interface, albeit with there being substantial engineering issues that remain before the potential of nano neural interfaces can be fully exploited. Within this framework, we review the qualitative breakthroughs and opportunities brought about by 3D vertical nanoelectrodes, and discuss the major limitations of current electrode designs with respect to rational and seamless cell-on-chip systems.

## Abstract 2

Developing electrophysiological platforms to capture electrical activities of neurons and exert modulatory stimuli lays the foundation for many neuroscience-related disciplines, including the neuron–machine interface, neuroprosthesis, and mapping of brain circuitry.

## Graphical abstract

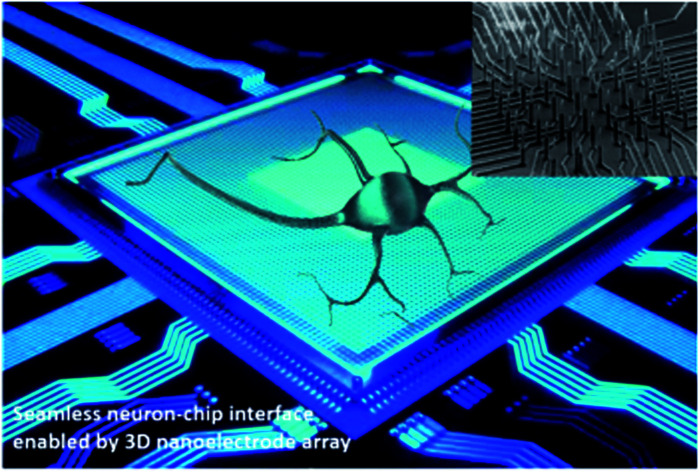

## Introduction

Electrogenic neurons, as the control units of most biological living beings, have great potential in advancing life technologies and artificial intelligence. In the central nervous system, neuronal networks are able to learn adaptively from environmental inputs, form cognitions, and carry memory storage. In the peripheral nervous system, neurons can sense a diversity of mechanical, chemical, and thermal stimuli, while delivering accurate controls through neuromuscular junctions for both long-range, high-strength and short-range, delicate motions. Moreover, the highly efficient transformation from chemical energy to ionic gradients allows a neuron to generate and transmit electric signals. Therefore, it has long been a major pursuit in neuroscience, bioengineering, and electrical engineering to develop seamless neural interfaces for probing, understanding, and modulating neural activities. And, despite advances in engineering large-scale electrophysiological approaches for *in vivo* applications,^1–7^ establishing neuronal interfaces at the cellular and subcellular levels *in vitro* is still imperative to answer the fundamental questions of how to achieve high-fidelity and reliable cell-to-chip communications.

At the implementation level, constructing a bridge between electronic devices and neurons requires the electrodes not only to have appropriate electrical properties for signal detection and/or current injection, but also to be able to adapt to the dynamic and fragile nature of cells. Conventional tools of intracellular micropipettes (*e.g.* patch clamps) and extracellular microelectrode arrays (MEAs) have intrinsically suffered from the trade-off between invasiveness and precision. Patch clamping often leads to severe cell damage within hours of electrode insertion, and is difficult to expand to multisite, parallel recordings. While planar MEAs impose no invasiveness to cells, the large dimension of electrode size (often close to or bigger than neuronal soma) and poor electrode–membrane seal make it difficult to reveal subcellular and subthreshold neuronal activities. Looking at nature, the nanoscale ion channels are the driving forces behind the great diversity of neuronal dynamics, which provide biomimetic inspiration to overcome this trade-off by shrinking the electrode dimensions to the nanoscale. Although a variety of nanostructures, such as planar nanowires and suspended nanoparticles,^8–13^ have been applied to neuronal recording and stimulation, a revolutionary breakthrough towards an organized and high-resolution neural interface has been enabled by 3D nanofabrication techniques. Vertical nanoelectrode arrays significantly reduce the projection area on the planar substrate to which they are bound without compromising the interfacial area they share with the local cell membrane, allowing for the realization of high-density fabrication and parallel recording from different compartments of single neurons. More importantly, their topography of 3D protrusions provides intimate electrode–membrane seals, drastically improving the reliability and fidelity of recording. In this way, nanoelectrode arrays not only induce sufficient invasiveness to ensure high-quality signals, but also cause minimal cellular damage even after electrical or optical poration. Therefore, these advantages that have arisen from scaling down to the nanoscale have provided new possibilities for solid-state electronics to “talk” to electrogenic neurons.

In this focused review, we will discuss the fundamental advantages and issues of *in vitro* nano neural electrodes towards the goal of accurate and rational cell–machine interfaces, primarily focusing on vertical nanoelectrode arrays that are most promising for large-scale, parallel neuronal interfaces. Specifically, we start with the neuronal computation process enabled by nanoscale ion channels and neuronal projections, as well as a brief overview on the electrical interface between nanoelectrodes and neurons. Next, we highlight the opportunities provided by nanoelectrodes regarding resolution, signal quality, intracellular access, and fabrication flexibility. And finally, we discuss the challenges of current *in vitro* nanoelectrode platforms from the system level of mapping of neuron electrical dynamics and constructing functional cell-on-chip systems.

## Nanoscale neural operation

### Ionic operation and neuronal computation

Despite the complexity and plasticity of large-scale neural networks, neuronal operations are still governed by the laws of thermodynamics, in which the interactions between chemical and electric potentials are the sole factors that control ionic transport. Various species of cations, such as Na^+^, K^+^, and Ca^2+^, serve as charge carriers for a neuron to generate transmembrane currents. Assisted by active ion transporters,^14^ a neuron can maintain constant gradients for each ionic species across its membrane (Table 1). At rest, for each permeable ionic species, its chemical concentration difference across the intracellular and extracellular spaces gives rise to an electric potential difference governed by the Nernst equation (eqn (1)), which tends to counterbalance the transmembrane concentration gradient. The result is an equilibrium where its chemical and electrical potentials are balanced:1
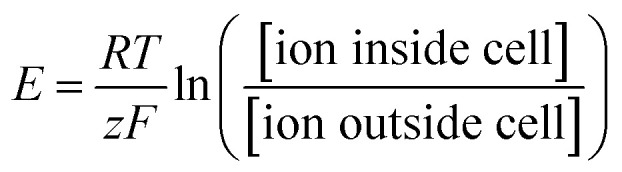


**Table 1:** Major charge carrier concentrations for typical mammalian neurons

Ion species	Intracellular concentration (mM)	Extracellular concentration (mM)
Na^+^	10	145
K^+^	140	5
Mg^2+^	0.5	2
Ca^2+^	10^−4^	2
Cl^−^	10	110

This Nernst potential is the driving force behind charge carrier transports across the neuronal membrane, and selective ion channels serve as gates to recruit certain ion species for an action potential and to regulate the temporal dynamics of ionic currents. For example, neuronal membrane at rest has very low permeability (non-zero) to Na^+^, thus the cytoplasm and extracellular fluid are nearly isolated systems for Na^+^. Although extracellular Na^+^ is both high in chemical potential and electric potential, the equilibrium potential of Na^+^ (defined by eqn (1)) has little effect on the resting membrane potential due to such separation. The activation of Na^+^ channels during the early stage of an action potential, once it occurs, brings two spaces into a single system where Na^+^ diffuses from the extracellular fluid to the cytoplasm, tending to reach its equilibrium electrochemical potential. Such an alternative electricity is a direct result of the neuron's contact-separate strategy in that it uses selective ion channels to switch the transports of its charge carriers at different stages of an action potential.

**Fig. 1:**
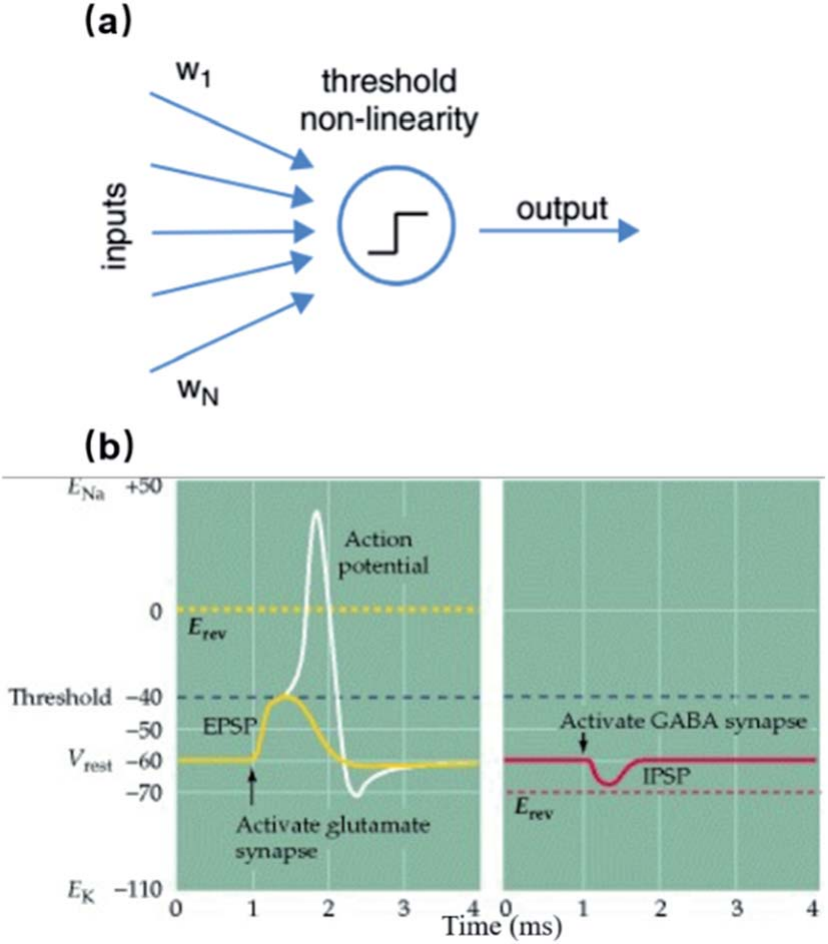
Neuronal computation process and subthreshold synaptic inputs. (a) The classic McCulloch–Pitts neuron performs a weighted sum of its synaptic inputs (each input *i* is multiplied by a synaptic weight *w*_*i*_), and then a threshold operation. Each incoming presynaptic signal produces a PSP at the postsynaptic terminal, which spreads passively to the cell body (spatial summation). The cell body will perform temporal summation of all of the PSPs from different synapses. If the resulting average PSP at the soma exceeds the potential threshold, an action potential will be fired. This figure has been adapted from ref. 15 with permission from Elsevier. (b) Examples of an excitatory post-synaptic potential (EPSP) and an inhibitive post-synaptic potential (IPSP). The reversal potential (dotted line) of an EPSP is more positive than the action potential threshold, increasing the probability of triggering an action potential. For IPSPs, the reversal potential is more negative than the threshold, producing an inhibitive effect on action potential generation. This figure has been adapted from ref. 16 with permission from Sinauer Associates.

At the cellular level, a single neuron is an independent computational unit and the generation of action potentials is just the result of its computation process. The post-synaptic potentials (PSPs) from distal dendrites, either excitatory or inhibitory, are integrated at the neuronal soma that determines if an action potential will be fired or not (Fig. 1a).^15^ Unlike the digital all-or-none feature of action potentials, these synaptic inputs are analog signals with different amplitudes and durations (Fig. 1b).^16^ More importantly, because of neurite migration and synaptic plasticity, PSPs are both spatial and temporal variants. Taking into account the non-uniform distributions of ion channels, these unique characteristics of neurons will certainly require an electrophysiological platform capable of high-resolution, long-term, and seamless recording/stimulation, if we want to govern the detailed neuronal dynamics.

### Nanoelectrode–neuron interface

**Fig. 2:**
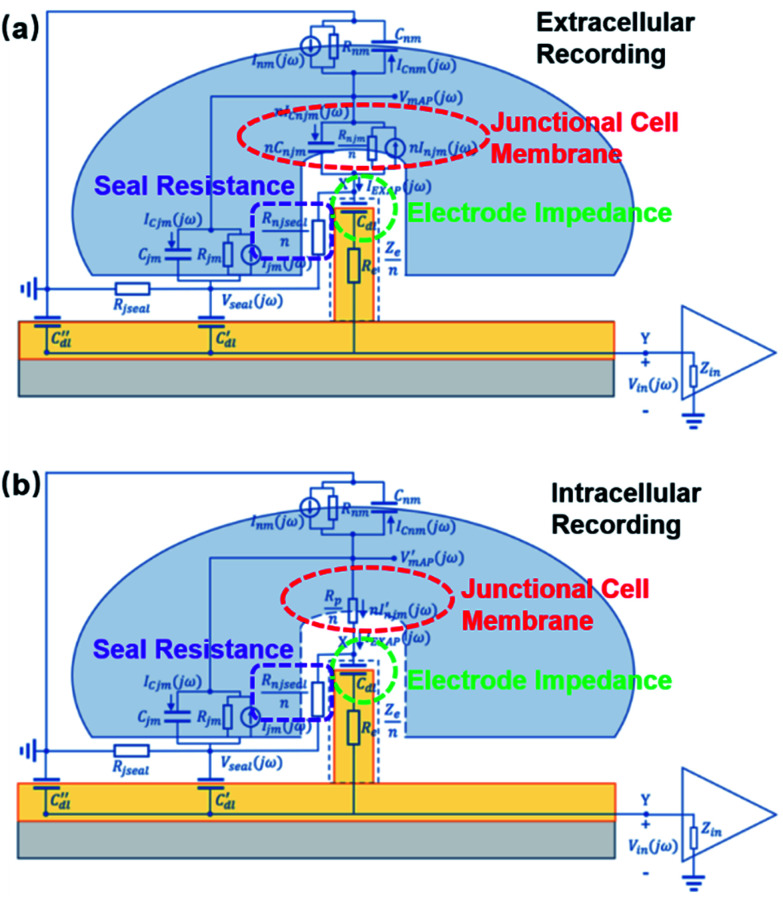
Equivalent electrical circuit models of a nanoelectrode–neuron interface during action potential recordings. Transmembrane ionic currents from junctional membrane (red) are the signal sources to be detected by the nanoelectrode. The nanoelectrode-electrolyte interface (green) is a passive capacitor representing the EDL. The adhesion between the electrode and junctional membrane is modeled as a seal resistance (purple), through which current leakage attenuates the amplitude of recorded signals. The equivalent circuits of *n* interconnected nanoelectrodes are shown. (a) Extracellular recording in which the transmembrane current through local ion channels is the signal source that alters the charge distribution in the EDL. (b) Intracellular recording in which the nanoelectrode gains electrical access to the cytoplasm. The internal potential of the neuron connects to the electrode through a pure resistor with its resistance determined by the pore size in the membrane (see *Intracellular Access Through Membrane Poration*). These figures have been adapted from ref. 17 with permission from IOP Publishing.

The recording/stimulation principle of 3D vertical nanoelectrodes is the same as planar MEAs. However, the small dimensions and vertical protrusions of nanoelectrodes can bring about significant improvements to some critical parameters. The equivalent circuit of a nanoelectrode–neuron interface is shown in Fig. 2, and a comprehensive discussion on recording mechanisms is covered in ref. 17. The nanoelectrode–electrolyte interface is an electric double layer (EDL) of capacitive nature during recording. Electrical activities of local membrane, in the form of transmembrane ionic current, cause charge redistribution in the EDL. The electrical potential variation caused by such a charge redistribution is recorded by the amplifying circuit. Because cell membrane spontaneously wraps around the nano-protrusions (like endocytosis), the resulting tight adhesion can greatly reduce the ionic cleft between the electrode and lipid membrane, supressing the current leakage through the seal resistance, *R*_seal_. Moreover, the membrane curvature is spontaneously formed without forced insertions, thus imposing minimum invasiveness and damage to the neuron. Since the junctional area is also at nanoscale, the signal source from the neuronal membrane is highly localized, allowing for the recovery of spatial distribution of the ion channels and membrane properties.

## Qualitative breakthroughs by nano probes

Even for animals with the seemingly simplest behaviors, there might be a rather complicated nervous system functioning in the background. In contrast to the rapid development in solid-state electronics, in which billions of transistors are integrated per chip, it took decades for scientists to resolve the complete connectivity of *C. elegan*'s nervous system, an invertebrate worm with only 280 neurons.^18^ As mentioned before, a single neuron is a complex computational device that receives inputs from multiple pre-synaptic terminals followed by neurocomputation to produce action potentials of various frequencies. Although the properties of ion channels have been extensively characterized using patch-clamp electrophysiology,^19,20^ their non-uniform spatial distribution and time variance make it challenging to decode the computation process. The situation becomes more frustrating for neural networks, where the variance of synapse location and time-dependent synaptic strength are involved. Therefore, to sketch the complete picture of nervous system operations, it is imperative, at the fundamental level, to first develop *in vitro* sensors with high resolution and long-term robustness to reveal the electrical activities of single neurons.

### Precise subcellular interrogation

Fundamentally more advanced than microelectrodes, nanoelectrodes are not only a matter of size-scaling, but a breakthrough regarding both the quantity and quality of information extracted from cells. MEAs, developed for the purpose of large scale and parallel neural recordings, are intrinsically limited by their large dimensions and defects at the neuronal interface. First of all, with nanoscale ion channels and submicron neurites and synapses, it is difficult for MEAs to pinpoint specific cellular compartments and extract localized information from plasma membrane, as the electrode size of 5–30 μm can only characterize the averaged electrical activities of the attached membrane. As a result, subcellular information reflecting the operation of nanoscale ion channels and submicron neurites and synapses is often attenuated or buried in the signals recorded from larger areas. Second, planar microelectrodes suffer from relatively weak electrode-cell coupling that originates from a 70–100 nm cleft between the cell membrane and solid-state probes.^24^ Such a cleft, filled with highly conductive electrolyte, contributes to most of the current leakage of the entire recording system.^25^ Although the topographical improvement using mushroom protrusions has greatly enhanced the membrane adhesion for cardiomyocytes and *Aplysia* neurons,^26^ the inevitable cell membrane deformation of micrometer size occupies a considerable amount of membrane area, making it difficult to simultaneously extract information from multiple sites of a small mammalian neuron.

**Fig. 3:**
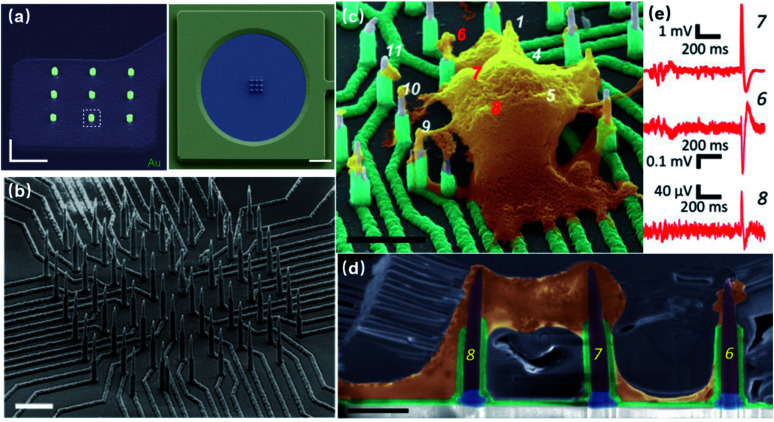
Interconnected and independent nanoelectrode arrays. (a) Scanning electron microscopy (SEM) images of interconnected nanopillar electrode arrays fabricated on a passivated microelectrode pad. The nanopillars are essentially a combined single recording unit, as they are electrically connected by the conductive pad underneath. Left, scale bar: 2 μm; right, scale bar: 10 μm. These figures have been adapted from ref. 21 (left) and ref. 22 (right) with permissions from Springer Nature. (b) SEM image of an 8 × 8 Si nanowire array, where each electrode can independently record neuronal signals. Scale bar: 3 μm. (c) The nanoelectrode array in (b) interfacing with a neuronal soma at different locations simultaneously. Scale bar: 4 μm. (d) Cross section of nanoelectrode–membrane interface showing intimate membrane engulfment. Scale bar: 2 μm. (e) Variation of signal shapes and amplitudes from different recording sites, allowing for detailed mapping of ionic current distributions. (b–e) have been adapted from ref. 23 with permission from the American Chemical Society.

The development of nanoelectrodes, especially vertical nanoprotrusions, allows probes to integrate with cell membrane at a much finer scale. This intrinsic feature, on the one hand makes it possible for nanoelectrodes to accommodate for the non-uniform distributions of ion channels on the neuronal membrane and to record potentials generated from local ionic currents, which may reveal detailed information on neural computation dynamics.^27^ Nanopillars, with a diameter of about 100 nm, can interface with only a small portion of the cell membrane, allowing for multiple detection at different sites from a cell. Moreover, nanowire field-effect transistors (FETs) have reached the 10 nm scale, approaching the dimensions of single ion channels^28^ (though this device has not achieved extracellular recordings from single ion channels). Early studies often fabricated multiple nanopillars on the same conductive pad, resulting in a loss of individuality, as they are electrically interconnected (Fig. 3a). However, the parallel probing advantage of nanoelectrodes has been realized by the breakthrough in high-density and independent nanowire arrays with a site-to-site spacing of only 750 nm,^23^ where each nanoprobe, as an independent unit, can acquire high-fidelity electrical signals from its local interface with the neuronal membrane (Fig. 3b). Such submicron-scale information from different sites (Fig. 3c–e), combined together, will be valuable for mapping the electrical properties of cell membrane and to understand the spatial and temporal mechanism of how neuronal signals are generated and transmitted.

It should be noted that the nanoscale resolution discussed above is limited to extracellular recordings because the potential change in the EDL relies on the transmembrane current. Once electrodes gain intracellular access, the recorded signals will reflect intracellular potentials that are affected by the space constant of cellular compartments.

### Enhancement of seal resistance

**Fig. 4:**
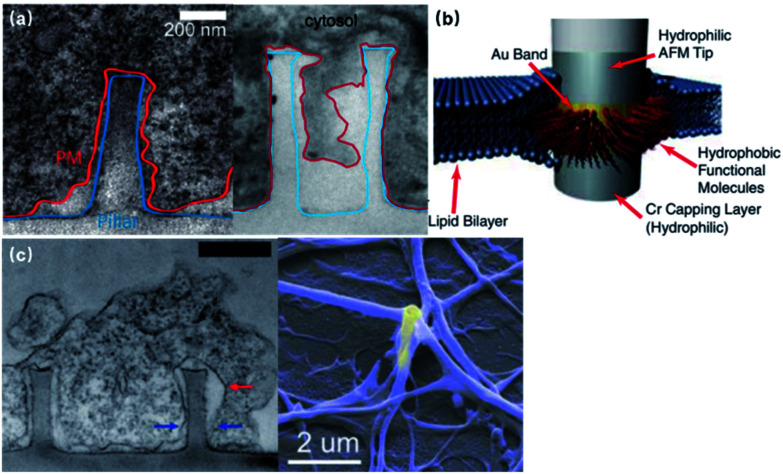
Seal resistance enhancement by membrane engulfment on 3D nanoprotrusions. (a) Left, transmission electron microscopy (TEM) image of the vertical cross-section of the intimate cell–nanopillar interface. The blue line indicates the border of the nanopillar and the red line indicates the cell's plasma membrane. This figure has been adapted from ref. 29 with permission from the American Chemical Society. Right, TEM vertical cross-section of a cardiomyocyte growing on top of a quartz nanotube showing that the bottom plasma membrane protrudes into the nanotube. This figure has been adapted from ref. 21 with permission from Springer Nature. (b) Nanopillar coated with a hydrophobic band for tight a GΩ seal. The 5–10 nm hydrophobic band is self-assembled from butanethiol on an Au band. The interactions between the hydrophobic band and cell membrane core produce a tight seal that is impermeable to charged ions, similar to the behavior of membrane proteins. This figure has been adapted from ref. 34 with permission from the National Academy of Sciences. (c) Left, vertical cross-section of large neurite engulfed nanopillars. The red arrow marks a cavity formed on the upper half of the nanopillar, while the blue arrows point out where the neurite is sealed tightly at the bottom of the nanopillar. Scale bar: 500 nm. This figure has been adapted from ref. 29 with permission from the American Chemical Society. Right, SEM image of neuronal processes enveloping a gold plasmonic 3D nanoelectrode. This figure has been adapted from ref. 37 with permission from the American Chemical Society.

The spontaneous membrane engulfment around nanoelectrodes provides an excellent seal for recording subthreshold membrane potentials. Various studies have characterized the plasma membrane deformation on vertical nanoprotrusions using fluorescence and electron microscopic techniques (Fig. 4a). Although the value of cleft thickness has shown substantial variance among devices, from less than 5 to 18 nm,^29,30^ there is still no doubt that their membrane attachment is much tighter than that of planar microelectrodes (*i.e.*, a 70–100 nm cleft), giving rise to a seal resistance of 80^29^ to 500 MΩ,^22^ which is a more than two orders of magnitude enhancement. Besides the morphologically tight adhesion, the membrane curvature caused by vertical nano-protrusions may also induce the aggregation of ion channels, increasing the local ionic current.^31,32^

Furthermore, treated using hydrophobic bands, nanopillars can even enhance their seal resistance to the GΩ range, which is comparable to that of patch-clamp seals (Fig. 4b).^33,34^ Despite the increased electrode impedance caused by the reduced surface area, the improvement in the membrane–electrode seal clearly overweighs the sacrifice of electrode impedance, which has been proved through both theoretical study^25^ and electrophysiological tests.^21–23,35,36^ More importantly, membrane engulfment was also observed at large neurites that are difficult to access by patch-clamps and microelectrode arrays (Fig. 4c),^29^ providing the promise for the parallel monitoring of dendrites, axons, and pre- and post-synaptic terminals, as these compartments play more significant roles than the soma regarding neural network plasticity.

### Intracellular access through membrane poration

**Fig. 5:**
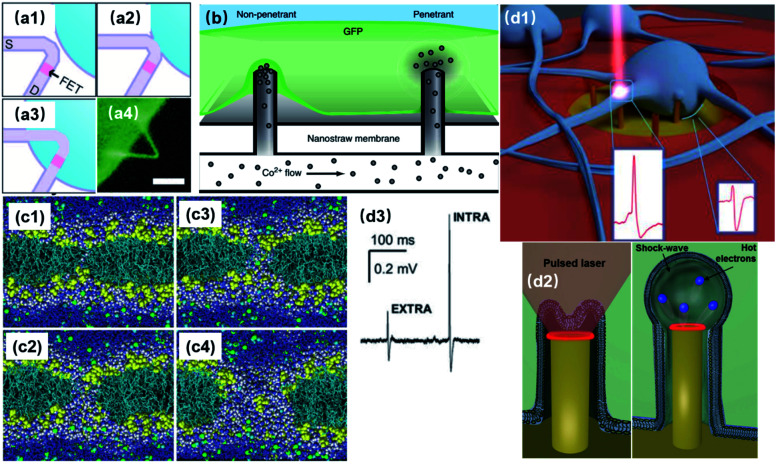
Intracellular access through localized membrane poration. (a1–a3) Schematics of a phospholipid-coated Si nanowire field-effect transistor (FET) probe being inserting into a cell. The process is similar to endocytosis because of the lipid coating and nanoscale dimension. (a4) False-color fluorescence image of a lipid-coated nanowire probe. Scale bar: 5 μm. These figures have been adapted from ref. 38 with permission from AAAS. (b) Spontaneous membrane penetration by vertical nanostraws, where ions can be delivered into the cytoplasm through the nanostraw hollow tunnel. This figure has been adapted from ref. 40 with permission from Springer Nature. (c) Simulation of the electroporation process. The lipid headgroups are shown in yellow, the chains in cyan, chloride ion space filling in green, sodium ions in cyan; water is shown in dark blue and white space filling in the interface region and the pores, and as dark blue bonds elsewhere. This figure has been adapted from ref. 41 with permission from BioMed Central Ltd. (d1) Schematic of plasmonic optoporation platform for neurons cultured on 3D nanoelectrodes. Low-power laser beams can selectively induce a plasmonic effect at individual electrodes to open pores at specific locations. This figure has been adapted from ref. 37 with permission from the American Chemical Society. (d2) The mechanism of plasmonic membrane poration. Hot electrons excited by laser irradiation induce a mechanical shock wave in water, rupturing the cell membrane. This figure has been adapted from ref. 42 with permission from Wiley. (d3) Recorded electric signals before (EXTRA) and after (INTRA) membrane poration. The significantly enhanced signal amplitude and intracellular-like signal shape indicate that electrode has gained intracellular access after poration. This figure has been adapted from ref. 37 with permission from the American Chemical Society.

Membrane poration can be conveniently applied to nanoelectrodes to directly record from the cytoplasm with higher accuracy and less invasiveness comparing to patch clamps and MEAs. By penetrating the cell membrane either spontaneously or artificially, intracellular signals will pass through a resistive interface to nanoelectrodes, rather than being “filtered” by the membrane capacitance (Fig. 2b). Also, due to the rather large dimensions of pore openings comparing to leaky K^+^ channels, the access resistance is significantly lowered, yielding a high signal-to-noise ratio (Fig. 5d3).

Unlike the invasive sharp electrodes that often lead to cell death after impalement, vertical nanostructures impose little damage on the lipid membrane. The sub-100 nm dimensions and high aspect ratio of the nanostructures induce endocytosis, allowing them to fuse into cells (Fig. 5a).^38^ With biomimetic surface modifications^38,39^ or hydrophobic coatings,^34^ the fluid leakage between the cytoplasm and extracellular medium can also be reduced significantly, making nanoscale penetration suitable for repeatable intracellular access over long-term recordings.

Spontaneous membrane penetration enabled by vertical nanoprotrusions was observed and widely applied in delivering biomolecules into the cytoplasm (Fig. 5b). Without external forces such as centrifuging or manual penetration, the high aspect-ratio of vertical nanowires can induce penetration of the cell membrane by gravity or adhesive force driven internalization after cell plating,^43,44^ which has been reported for various cell lines, primary neurons, fibroblasts, and immune cells.^45–50^ Theoretically, for nanoelectrode recordings, spontaneous intracellular access has the advantage of a stable electrode–neuron interface in which internalized electrodes can maintain their cytoplasm access for a long period of time.^40^ Moreover, excluding external forces will reduce the probability of cell damage. However, the performance of this approach has not yet met the requirements of parallel intracellular electrophysiology. The bottleneck lies in the unclear penetration mechanism and low penetration rate of the nanowires. Although surface modification with cell adhesion molecules (CAMs) or cell penetrating pipettes (CPPs) have enhanced the internalization efficiency to 15%,^44,51^ it is still not affordable for neuronal recordings, since a nanoelectrode array has much fewer probes than relatively simple drug delivery platforms. In addition, a systematic study using nanopillars, nanocones, and sharp nanopillars has revealed that vertical nanostructures spontaneously penetrate the cellular membrane to form a steady intracellular coupling only in rare cases, and suggested that most spontaneous penetrations might occur only during the initial hours of cell plating with membrane resealing afterwards.^53^ Therefore, it is important to address the significant issues in terms of both mechanism and application before spontaneous poration can be exploited further for nano neural electrodes.

On the other hand, artificially induced membrane poration through electrical or optical approaches has been well applied to intracellular recordings. In electroporation, cell membrane can be ruptured under a strong electric field applied at the nanoelectrode tip, allowing for significant improvement in the signal amplitude and intracellular-like shapes. The mechanism of this process was investigated by computer simulations. Specifically, water defects, caused by the interaction of water dipoles with the electric field gradient at the water/lipid interface, are significantly promoted by the external electric field. As a result, water can penetrate the lipid bilayer from both sides, leading to pore formation (Fig. 5c).^41^ However, since electroporation has to be conducted by the same electrodes as used for the recordings, the switching between pulse injection and recording modes will inevitably result in a blind recording period caused by overcharging. Additionally, the number, size,^21,54^ and distribution of membrane pores from electroporation are difficult to predict and characterize.^55^ To address these issues, the combination of vertical nanoelectrodes and plasmonic optoporation was developed for opening transient pores at the tips of nanopillars (Fig. 5d1).^37^ Upon irradiation by laser pulses, the Au surface of the nanoelectrode generates highly energetic electrons (hot electrons) into the water conduction band, which induce a chain reaction of photon absorption and the release of more hot electrons. Eventually, water molecules are accelerated by hot electron impact, producing a mechanical shock wave that ruptures the cell membrane (Fig. 5d2).^42^ So far, the electrode material has been limited to Au due to its low energy threshold (3.7–2.2 eV) for hot electron excitation^42,56^ at the solid–water interface. Comparing to electroporation, this approach also has the advantage of localized pore formation, in addition to continuous recording. Because of its essence of mechanical nanoshockwaves, plasmonic optoporation is likely to produce single pores that are highly localized and easier to modulate. Considering that the lipid membrane reseals after poration, a single and localized pore could make the recorded signals better in terms of fidelity and consistency than a group of sparsely distributed pores. This might also be the reason for the prolonged period of intracellular access of up to 80 min,^37^ as it takes more time for the cell membrane to reseal a large pore than many small pores.

For both electroporation and optoporation, the recording window is relatively short (10–80 min, depending on the poration method and parameters). However, nanoelectrode penetration is a highly local process that has little impact on the adjacent membrane as well as seal resistance. In fact, the cell membrane spontaneously reseals after the recording window. Thus, the poration could be repeatedly performed (once every several hours) over a long-term cell culture. Although this repeated sampling is not a continuous recording, considering that the neuronal long-term plasticity (learning and memory) is a much slower process, repeated intracellular access with nanoelectrodes still holds great promise for studying long-term neural network dynamics.

### Fabrication of nanoelectrodes

**Fig. 6:**
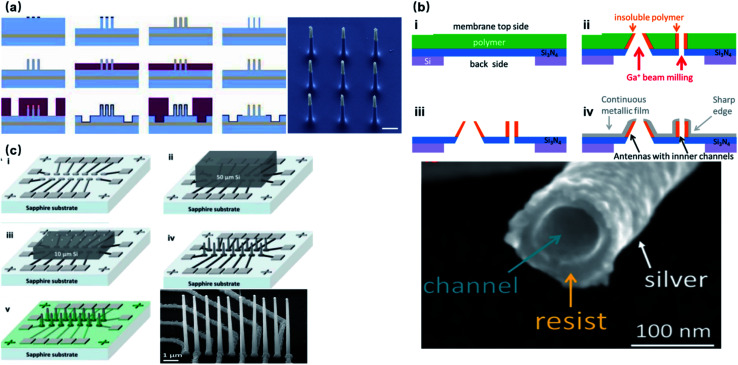
Top-down fabrication of vertical nanoelectrodes. (a) Si nanowires from oxide thinning and wet etching. The diameter of the Si nanowire is 600 nm after lithography and etching. The following thermal oxidation transforms Si to SiO_2_. After etching the SiO_2_, the nanowire diameter is reduced to 150 nm. This figure has been adapted from ref. 22 with permission from Springer Nature. (b) Metal nanotubes from secondary electron FIB milling. A Ga^+^ beam is used to define the hollow structures from the underside of the Si_3_N_4_ substrate. Secondary electrons from Ga^+^-polymer interactions expose the nearby resist, and the high electron doses and the resist heating will turn the resist tone from positive to negative. Thus, the exposed structures stay insoluble during developing, serving as cores for the metal coatings afterwards. This figure has been adapted from ref. 52 with permission from the American Chemical Society. (c) High-throughput electrode fabrication from thermal wafer bonding, allowing for individual nanowires to be registered precisely over underlying metal leads. Conventional lithography of the Ni layer first defines the electrode leads, wirings, and contact pads. A thin Si wafer is then thermally bonded to the Ni layer through nickel silicidation at 400 °C (NiSi formed), followed by e-beam lithography and reactive ion etching on Si to produce the vertical nanoelectrodes. This figure has been adapted from ref. 23 with permission from the American Chemical Society.

Most substrate-bound vertical nanoelectrodes with diameters of 100–200 nm can be either fabricated top-down from bulk materials or bottom-up through crystal growth or deposition. So far, fabrication techniques have been developed for only doped semiconductors (Si) and noble metals (Pt, Au) due to their compatibility with photo- and e-beam lithography as well as chemical and physical etching. The top-down process has been widely used for metal-coated semiconductor nanopillars and nanotubes, in which high-resolution lithography was first applied to define the diameters of electrodes on photoresists, followed by reactive ion etching to remove uncovered material and leave out vertical nanostructures of high aspect-ratios. Additionally, since the top-down approach is a well-established process in dealing with semiconductors, it promotes innovative improvements to achieve various electrode geometries, smaller diameters, and higher densities. For example, the diameter of Si nanowire electrodes was reduced from 600 to 150 nm by thermal oxide thinning, based on that Si can be thermally oxidized to SiO_2_, which can then be selectively etched by HF (Fig. 6a).^22^ Fabrication of hollow nanotubes exploited the tone-inversion of photoresists by secondary electrons during focused ion-beam (FIB) milling (Fig. 6b), resulting in a cylinder wall that could not be removed by developers.^52^ Plus, the density of independent nanoelectrodes was greatly enhanced by thermally bonding a Si wafer onto patterned metal leads before e-beam lithography and plasma etching (Fig. 6c).^23^

**Fig. 7:**
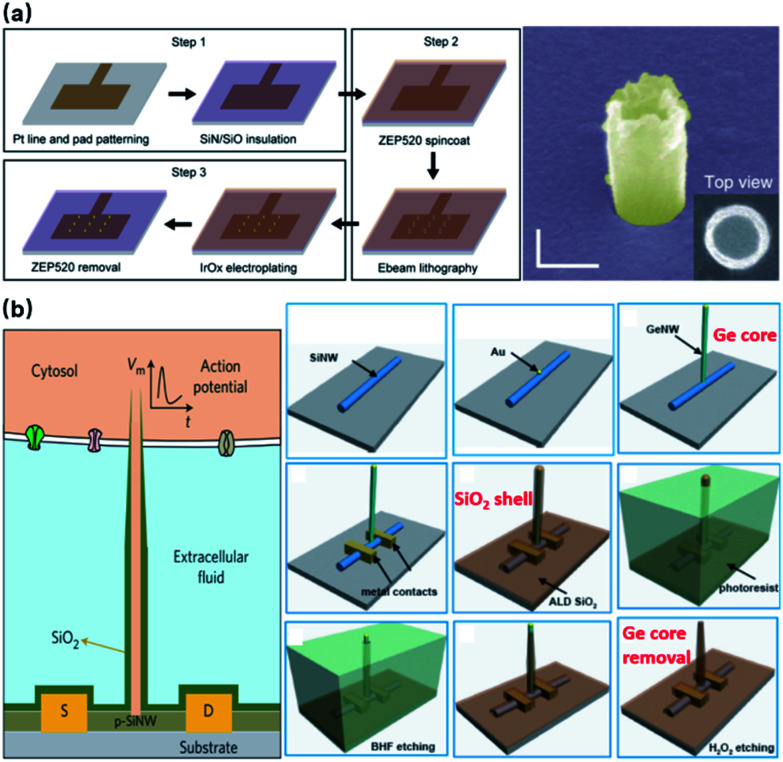
Bottom-up fabrication of vertical nanoelectrodes. (a) IrOx nanoelectrodes from electrochemical deposition. IrOx nanoprotrusions are electroplated on the pre-defined Pt nano patterns. This figure has been adapted from ref. 21 with permission from Springer Nature. (b) Schematic, fabrication, and SEM image of Si nanowire FETs from vapor–liquid–solid crystal growth. The inner core of the vertical nanotube is Ge nanowire grown through VLS on a 100 nm Au nanodot (produced by lithography on a lying-down Si nanowire). The entire device is then uniformly coated with SiO_2_ to form the outer shell of the nanotube. Finally, the Ge core is selectively removed by H_2_O_2_, yielding a hollow structure where ionic fluid makes contact with the lying-down Si nanowire. Therefore, intracellular electrical signals (as gate voltage) are able to modulate the current passing through the Si nanowire (from source to drain). This figure has been adapted from ref. 59 with permission from Springer Nature.

Noble metals of Pt and Au have excelled in microelectrodes because of their inertness in biological fluids and excellent conductivity, rendering robust neural interfaces for long-term sensing. For nanoelectrodes, however, metals are highly resistant to plasma etching, and it would be expensive and unreliable to use conventional lift-off processes for high-aspect-ratio protrusions that are 1–3 μm in height. Therefore, the bottom-up approach by selective deposition has been mostly adopted for metal nanoelectrodes. Specifically, nanoscale holes can be milled on a layer of photoresist by e-beam lithography, followed by electrochemical deposition or FIB-assisted deposition to create the protrusion structure (Fig. 7a).^21^ In comparison to semiconductor-based processes, bottom-up deposition involves much fewer steps that greatly reduce fabrication failure. Moreover, it is insensitive to substrates so that transparent glass and quartz can conveniently serve as substrates for cell observation under inverted microscopes. However, because of the limited maneuverability of noble metals at the nanoscale, bottom-up fabricated electrodes are often pillar-shaped without sharpened tips (like Si electrodes), which may more or less affect the cell membrane–electrode interface due to their restrained bending ability under the pulling force from the cell membrane and the larger curvature at the electrode tips.^57^ Another factor that makes semiconductor electrodes more popular is their compatibility with CMOS (complementary metal-oxide-semiconductor) technologies that integrate on-chip amplifiers in the vicinity of electrode probes, significantly reducing the amplitude loss by eliminating the stray capacitance.^58^

Besides electrode-based nanoprobes, bottom-up crystal growth through vapor–liquid–solid (VLS) deposition is also the most critical process in the fabrication of nanowire field-effect transistors (NWFETs).^38,59–61^ Composed of three terminals: source (S), drain (D), and gate (G), an FET's charge carrier density in the S–D channel is modulated by the voltage applied on the gate, thus resulting in a conductance controlled by the gate. During neural recordings, the alternating electrical potential from a neuron serves as the gate voltage that modulates the S–D current (Fig. 7b). The original neuronal potentials can then be recovered by fitting this current into the known transconductance characteristics of the FET. Despite their unique structure, the working principles of NWFETs are not essentially different from those of vertical nanoelectrodes, as both devices operate upon capacitive charge redistribution at the solid–electrolyte interface. Instead of transmitting signals to amplifiers through metallic wirings, FETs function as amplifying units integrated directly with EDL capacitance, thus eliminating the stray capacitance and wiring resistance that compromise the signal-to-noise ratio.^58^

## Dilemmas related to seamless neural-chip interface

### *In situ* characterization of the electrode–neuron seal

Neuronal signals acquired by nanoelectrodes are strongly affected by the seal resistance, *R*_seal_, due to high electrode impedance. More importantly, it is imperative to measure the *R*_seal_ for each electrode in order to recover a neuron's actual electrical activities. Indeed, the tight adhesion between the cell membrane and vertical nanostructures yields detectable intracellular-like signals after membrane poration. However, interfacing with different neuronal compartments of different dimensions, each electrode will possess a unique *R*_seal_, causing substantial variance across electrodes. Moreover, neuronal migration may also contribute to the time-variation of *R*_seal_. Apparently, if this critical parameter is not available or well characterized, the quantitative information of the recorded data will be lost.

**Fig. 8:**
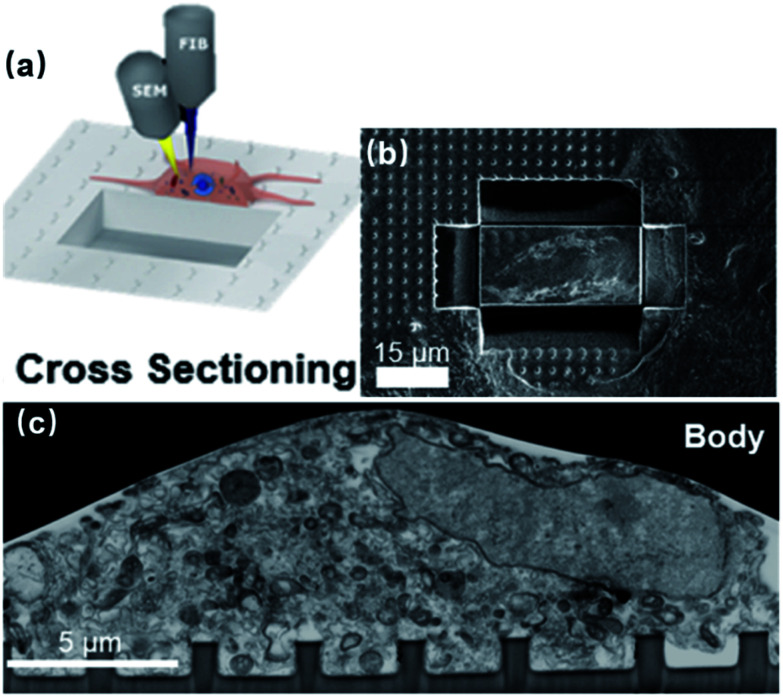
(a) Schematics and (b) experimental results of using FIB milling to cut trenches through the cell and the substrate and open up the interface. The FIB provides a high-energy Ga^+^ beam to cut through the sample and open up a vertical surface. Meanwhile, SEM conducts *in situ* imaging of the cross section. (c) FIB-SEM image of the neuronal body on a line of nanopillars. These figures have been adapted from ref. 63 with permission from the American Chemical Society.

A variety of methods have been developed to topographically characterize the membrane-surface gaps, which have been comprehensively covered in ref. 62. In general, imaging of the highest resolution can be achieved using TEM, where the cell-on-chip devices are thin-sliced before observation (Fig. 4a).^29,30^ Alternatively, FIB and SEM (FIB-SEM) can target the biointerface at any desired locations through FIB milling (Fig. 8),^63,64^ which greatly improves the technical flexibility over that of TEM. However, electron microscopy is a destructive process that can only be conducted at the final stage, as the cells have to be killed during sample preparation. On the other hand, live cell imagings, such as through fluorescent confocal microscopy^65,66^ and curvature-sensing of proteins,^67–70^ can qualitatively reveal the membrane engulfment around the nanopillars but cannot provide quantitative information on the seal resistance.

Despite the lack of techniques for *in situ* characterization, the membrane-surface seal can still be estimated from electrical models. However, the values of *R*_seal_ were significantly different among the different devices, from tens of MΩ to GΩ. Typically, these estimations were obtained by fitting the recorded waveforms into the electrical model of the electrode–neuron interface.^23,35^ For example, in an equivalent circuit model, the electrode impedance, stray capacitance, and amplifier input impedance can be either measured or estimated with reasonable accuracy. Given the signals generated by the target cell, the *R*_seal_ value can be estimated by sweeping until the simulation results fit well with the recorded data. However, in order to calculate *R*_seal_, the neuronal signal sources, which are supposed to be revealed by nanoelectrodes, are assumptively predefined. This approach might work well when recording action potentials with known waveforms, but when it comes to subthreshold signals from neurites, it will no longer be valid, as there are no predefined signals available to calibrate *R*_seal_. Therefore, for nanoelectrodes to work independently as robust neural interfaces, it is important to develop *in situ* measurement approaches.

### Bidirectional recording and modulation

High-fidelity bidirectional communication is critical for accurate and intelligent cell-on-chip systems and brain–machine interfaces, as neural probes should not only detect neural activities but also exert control over neural networks. For nanoelectrodes based on a solid–electrolyte interface, the capacitive nature of charge transport makes the recording quality more vulnerable to poor seal resistance. For a typical nanoelectrode with a 1 pF surface capacitance, its impedance is about 150 MΩ at 1 kHz, and will increase significantly for slower changing signals. Since such an impedance is comparable or higher than the seal resistance (100–500 MΩ), both the amplitude and shape of recorded signals will be affected substantially by the electrode–membrane seal. Moreover, low-frequency field potentials may have important links to the brain's perception, motion, and memory,^71,72^ but will be severely attenuated by the high impedance of nanoelectrodes. Additionally, considering the missing approach to accurately measure seal resistances *in situ*, the fidelity of acquired data has to be put under question.

On the other hand, this issue becomes more problematic for neural modulation purposes. The small surface area of nanoelectrodes results in an extremely low charge delivery capacity, which might be able to inject miniature post-synaptic currents (mPSCs, 10–20 pA amplitude, 10 ms duration^73^) but not simulate presynaptic spikes to evoke excitatory post-synaptic currents (eEPSCs, 0.1–0.2 nA amplitude, more than 50 ms duration^74^). Although larger current injection can be realized by breaking into the faradaic regime,^22^ the electrochemical reactions will inevitably cause electrode degradation and even water electrolysis for polarizable electrodes such as Au or Pt. Additionally, the slow recovery/discharging process after stimulation makes it difficult to immediately switch an electrode from stimulation to recording mode.

Direct ionic access with a resistive nature has the potential solution to these intrinsic constraints of solid–electrolyte interfaces. For example, conventional electrophysiological technology using fluid-filled micropipettes is capable of both signal acquisition and current injection at the same time. Despite major drawbacks in terms of destructive membrane penetration, fluid diffusion, and limited parallel access, the direct ionic interface established between the fluid-filled micropipettes and neuronal cytoplasm allows them to yield reliable, high-fidelity, and consistent results, due to their low access resistance (about 25 MΩ for sharp electrodes with a 10 nm tip). More importantly, combined with non-polarizable Ag/AgCl electrodes and a Wheatstone Bridge circuit, a micropipette electrode in current-clamp mode is able to implement simultaneous stimulation and recording.^75^

Such a concept was partially realized using nanotube intracellular probes made from chemically grown Si nanowires (Fig. 7b).^28,59^ With FETs integrated at the bottom of nanotubes, this bioelectronic probe provides high-resolution intracellular mapping of electrogenic cells. Yet the use of FETs as an interface also precluded the compatibility for current injection. Surprisingly, the breakthrough in nanoscale ionic neural interface occurred mainly in the field of drug delivery, where vertical nanostraws (hollow nanotubes) were used to impale cell membrane, allowing for the diffusion of molecules into the cytoplasm (Fig. 5b).^40,76–80^ Specifically designed for drug delivery, the nanostraws in these devices are not individually addressable. In addition, the unclear mechanism of spontaneous membrane penetration gives rise to unstable and low percentage of cytoplasm access. Despite their current shortcomings, the success of nanostraw fluid platforms and nanotube FETs still demonstrated that the ionic nano-neural electrophysiology is fundamentally and technically feasible, providing great promise for future improvements in engineering.

### Rational alignment with neurites and synapses

**Fig. 9:**
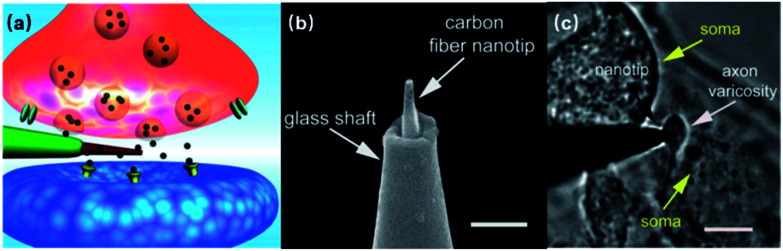
Carbon fiber nanoelectrode mounted on a glass micropipette for sensing synaptic events. (a) Schematic of a nanoelectrode tip inside a synapse. (b) SEM image of the CFNE. A carbon fiber etched down to 50–200 nm (diameter) is inserted into a pre-pulled glass micropipette. Scale bar: 1 μm. (c) Bright-field microscopy image showing the nanotip inside a synapse between a varicosity of a superior cervical ganglion (SCG) sympathetic neuron and the soma of another SCG neuron. Scale bar: 5 μm. These figures have been adapted from ref. 84 with permission from Wiley.

Despite the extraordinary signal acquisition performance of 3D nanoelectrodes, these devices should not be limited to the vision of capturing intracellular-like signals or gaining larger amplitudes, and a lot of engineering needs to be applied to fully exploit their potential for precise and parallel neural interfacing. If we treat a neuron as a system, its dendrites, axons, and synapses are where the ionic current inputs, outputs, and intercellular signal passage take place. In particular, it is the temporal and spatial diversity of chemical synapses that give rise to the plasticity of neural networks. Both excitatory and inhibitory synapses can exist on the same neuron, and the synaptic strength can be influenced by Hebbian plasticity^81^ or heterosynaptic modulation.^82^ These critical but small neuronal features at the 1–2 μm dimensions, although having been qualitatively studied using biological approaches,^83^ still need to be quantified by electrophysiology in order to establish interfaces with modern electronic chips. Li *et al.* have demonstrated that carbon fiber nanometric electrodes (CFNEs) can accurately access individual synapses and monitor the dynamics of neurotransmitter fluxes^84^ (Fig. 9), which suggests that nanoelectrodes hold great promise in hacking the neurites and synapses. However, CFNEs are mounted on glass micropipettes operated through a conventional patch-clamp setup, eliminating their potential for large-scale parallel recording. For vertical nanoelectrode arrays, however, the question still remains as to how to precisely access to the critical neuronal compartments while maintaining comprehensive coverage of the entire neuron. Therefore, engineering efforts in the areas of cell manipulation, neurite guidance, and nanoelectrode alignment are to be expected in the future to functionally integrate nanoelectrode arrays with neuronal circuits.

## Conclusions

The advancement of nano neural electrodes is a joint effort involving nanofabrication, neuroscience, electronic engineering, and biophysics. Behind this development lies the rationale of matching to the nanoscale neuronal compartments (*i.e.*, local ion channels, synapses, and neurites) that are critical to information processing. Ideally, neural interfaces should be able to selectively monitor the electrical activities of neurons without inducing acute damage or substantially altering the natural status of the neurons. For 3D vertical nanoelectrodes, scaling down the electrode sizes to less than 200 nm has caused minimal membrane deformation (relative to the total area), while enabling precise electrical neuronal interfaces at the subcellular level. Arranging the nanoprotrusions into individually addressable arrays further opens up the possibility of parallel, large-scale neuronal recording without labor-intensive manipulations.

Besides the rationale of accurate probing, organized vertical nanoelectrode arrays are more prominent over other nanoscale interfaces (such as dispersed nanoparticles and nanowires,^85^ and lying-down nanowires^86^), because the unique topography of substrate-bound vertical nanostructures addresses two significant barriers towards robust neuron-electronic integration: membrane–electrode seal and reliable intracellular access. The spontaneous membrane engulfment greatly reduces the current leakage through the seal, ensuring a good signal-to-noise ratio. And, membrane poration *via* electrical or optical approaches can be realized with low power injection, high repeatability, and membrane recovery. Overcoming these fundamental issues has made vertical nanoelectrodes a promising platform for neuron-chip communications.

Nevertheless, several key challenges still need to be addressed to push this technology further towards functional cell-on-chip systems. As mentioned in the remaining dilemmas, the prerequisite for understanding and decoding neuronal computation from recorded data is awareness of the recording conditions. The lack of approaches for characterizing the seal resistance *in situ* will inevitably make the recorded signals less trustworthy. On the other hand, the high electrode impedance from the EDL capacitance has put an intrinsic limitation on both recording and stimulation, which might be resolved by switching to direct ionic interfaces. Moreover, regarding device functionality, aligning nanoelectrodes with neurites and synapses needs to be addressed in the future to fully exploit the nanoscale advantages.

## Conflicts of interest

There are no conflicts to declare.

## Supplementary Material
